# Tonotopic reorganization and spontaneous firing in inferior colliculus during both short and long recovery periods after noise overexposure

**DOI:** 10.1186/1423-0127-20-91

**Published:** 2013-12-09

**Authors:** Feng Wang, Li Zuo, Bo Hong, Dongyi Han, Ethan M Range, Lingyun Zhao, Yanan Sui, Weiwei Guo, Liangfa Liu

**Affiliations:** 1 Department of Otolaryngology, The First Affiliated Hospital of Chinese PLA General Hospital, Beijing 100048, China; 2 Radiologic Sciences and Respiratory Therapy Division, School of Health and Rehabilitation Sciences, The Ohio State University College of Medicine, The Ohio State University Wexner Medical Center, Columbus, OH 43210, USA; 3 Department of Biological Sciences, Molecular Physiology and Biophysics Laboratory, Oakland University, Rochester, MI 48309, USA; 4 Department of Biomedical Engineering, School of Medicine, Tsinghua University, Beijing 100084, China; 5 Department of Otolaryngology-Head and Neck Surgery, Chinese PLA General Hospital, Beijing 100853, China; 6 Department of Otolaryngology, Beijing Friendship Hospital, Capital Medical University, Beijing 100050, China

**Keywords:** Cochlea, Noise, Tinnitus, Hair cells, Inferior colliculus

## Abstract

**Background:**

Noise induced injury of the cochlea causes shifts in activation thresholds and changes of frequency response in the inferior colliculus (IC). Noise overexposure also induces pathological changes in the cochlea, and is highly correlated to hearing loss. However, the underlying mechanism has not been fully elucidated. In this study, we hypothesized that overexposure to noise induces substantial electrophysiological changes in the IC of guinea pigs.

**Results:**

During the noise exposure experiment, the animals were undergoing a bilateral exposure to noise. Additionally, various techniques were employed including confocal microscopy for the detection of cochlea hair cells and single neuron recording for spontaneous firing activity measurement. There were alterations among three types of frequency response area (FRA) from sound pressure levels, including V-, M-, and N-types. Our results indicate that overexposure to noise generates different patterns in the FRAs. Following a short recovery (one day after the noise treatment), the percentage of V-type FRAs considerably decreased, whereas the percentage of M-types increased. This was often caused by a notch in the frequency response that occurred at 4 kHz (noise frequency). Following a long recovery from noise exposure (11–21 days), the percentage of V-types resumed to a normal level, but the portion of M-types remained high. Interestingly, the spontaneous firing in the IC was enhanced in both short and long recovery groups.

**Conclusion:**

Our data suggest that noise overexposure changes the pattern of the FRAs and stimulates spontaneous firing in the IC in a unique way, which may likely relate to the mechanism of tinnitus.

## Background

Tinnitus is a condition normally associated with hearing loss, and involves the perception of sound without the corresponding stimulus [1-3]. The reorganization of the frequency topographic map (FTM) has shown to be a direct outcome from hearing loss [4-7]. This could be associated with an increase of spontaneous firing in the brain auditory center [8,9]. It is accepted that such reorganization and spontaneous firing [1,3,10] are likely involved in the mechanism of tinnitus.

Previous studies have determined that the auditory system contains a topographic representation of tone frequency. The inner hair cells of the cochlea are tonotopically organized, resulting in each inner hair cell having tonotopic connections, through its associated ganglion cells, to cells in the cochlear nucleus [11]. Interestingly, topographic plasticity in the adult central auditory system has been well documented using a wide variety of techniques that result in partial ablation within the periphery of the auditory system. These techniques include mechanical disruption of the organ of Corti [5,6] and spiral ganglion [7,12], administration of cochleotoxic drugs [4,13,14], and exposure to high intensity sounds [15,16].

The documented changes in the inferior colliculus (IC) included: expanded lesion-edge frequency representations, shifts in characteristic frequencies that cause the expanded lesion edge frequency representations within affected frequency-response areas (FRA) [7,12], reduced levels of sideband inhibition at the particular frequency causing hearing loss [12,15,17], and reduced inhibition response from multi-units [4]. The changes within the IC occurred immediately after lesions developed and continued for several weeks to several months. For example, hearing loss greater than 10 dB, induced by noise exposure, caused instantaneous changes in the primary auditory cortex [15].

At a single-unit level, tonotopic re-organization of the IC is related to a shift in the characteristic frequency (CF). The shift included a distinct staircase pattern [18] along the dorsoventral axis. Following noise exposure, the original stepwise frequency-depth function changed significantly. In some distortions, only two or three “steps” appeared. The V-type, which is most common in the IC [7,12,19], translated to double peaks or multi-peaks in the FRA. Non V-type FRAs included two or three frequency peaks [16]. The CF thresholds significantly increased with the time following noise exposure, partially accounting for the occurrence of tinnitus [15]. Furthermore, previous reports have shown that auditory neural hyperactivity appears in the auditory cortex, IC, and cochlear nucleus, occurring simultaneously with tinnitus [1,20]. Yet, the mechanisms of tinnitus, which are associated with hearing loss, have not been clearly identified in these models.

To understand these complex mechanisms associated with tinnitus, narrow-band noise was utilized to induce cochlear damage in guinea pigs. We have measured the FRAs, spontaneous firing of single units in the IC, and cochlear damage in a single cell level, following noise overexposure of one day (short recovery) and 11–21 days (long recovery), respectively. Our data provide a new perspective in understanding the mechanism of noise induced tinnitus.

## Methods

All procedures were approved by the Department of Otolaryngology at The First Affiliated Hospital of Chinese PLA General Hospital and followed all required guidelines. Care and use of the animals in this study was also approved by the Institutional Animal Care and Use Committee of the Chinese PLA General Hospital. Nineteen healthy guinea pigs of both sexes with normal auricular reflex, weighing between 227 g and 525 g were used. The animals were randomly divided into three groups. Short recovery group (7 animals) was evaluated 24 hours after noise exposure and long recovery group was evaluated 11–21 days after exposure (6 animals). In the control group, six healthy animals were used. This timeframe (11–21 days) was consistent with previous studies, which showed that compound action potential (CAP) thresholds were fractionally recovered two weeks after noise-exposure [3,21,22].

### ABR thresholds

The auditory brainstem response (ABR) was also measured. Specifically, guinea pigs were anesthetized with xylazine (0.1 mg/kg) and ketamine (30 mg/kg). Subdermal electrodes were inserted at the vertex and pinna. A speaker was positioned directly above the mid-line of the animal’s head at a height of 12 cm. Tone-pips (0.5-ms on rise/fall time, at 30/sec) at 2, 4, 8, and 16 kHz were used as sound stimuli. The response signal was amplified, filtered, and averaged using the Intelligent Hearing System software (ISEN Tech and Trading, Ltd. Beijing, China) [23].

### Conditions for noise exposure

During this procedure, the animals were not anesthetized and thus were fully awake, undergoing a bilateral exposure to noise, which was produced from a signal machine (Model 33220A, Agilent Technologies, Inc., UK), intensified by a power amplifier (Model 2706, Brüel & Kjær Sound & Vibration Measurement A/S, Denmark), and transmitted to the surrounding speakers (Model D1002, Beijing First Radio Equipment Factory, Beijing, China). The acoustic overexposure stimulus was a 1/3 octave narrow band noise (i.e. white noise filtered by 1/3 octave band filter) at 4 kHz and 120 dB, measured by a sound level meter (Model 2209, Brüel and Kjær Sound and Vibration Measurement A/S, Denmark), and lasted 4 hours within a sound insulated room. The speakers were placed 4 cm in front of the head of an unrestrained guinea pig within small cells in a subdivided cage (1 animal/cell). Noise calibration, targeting SPL, was performed immediately before each exposure session. It should be noted that sound pressure levels varied by < 1 dB across the cages.

### Surgery procedure

Before the surgery, a dosage of 0.7 ml/100 g urethane was injected intraperitoneally for anesthetic purposes. The level of anesthesia was monitored in accordance with breathing state and blink reflex during the experiment. If the blink reflex occurred, one third of the concentration of the first dose of urethane was added to maintain a state of unconsciousness. The animals were given a preoperative subcutaneous injection of a dosage of 0.05 ml/100 g atropine to reduce respiratory secretion and to prevent respiratory tract obstruction during the experiment. Oxygen was administered through a customized mask while conducting the experiment. The animal body temperature was maintained by an electric blanket (Model 050100C0001B, FHC Inc. Bowdoin, ME, USA). After the guinea pigs were anesthetized, they were placed in the stereotaxic apparatus. The scalp, temporal muscle, and connective tissue were removed and the skull was exposed. A 10 cm long flat head screw was fixed on the top of the skull using glue and reinforced by dental cement (Model: 200805, Dental Division in Shanghai Medical Equipment, Ltd., Shanghai, China). An area which is 2 mm behind lambda, and 2.5 mm to the right side of the midline, was marked as a circle center with a radius of 2.5 mm. The skull included in the radius of the circle was removed by a micro-drill and the dura mater was removed.

### Generation of stimuli

Digital signals generated with 200 kHz sampling rate were used as the sound stimuli. After analog conversion (RX6; Tucker-Davis Technologies, USA) [24,25], the stimuli were applied to the anesthetized animals through the speakers (Electrostatic speaker 1, TDT Co., USA) which were placed next to each ear 20 cm from lambda in a free field. The rise/fall time of pure tone was 5 ms [17]. The stimulation sequences included two steps: 1) A sweep sequence was used for the determination of CF and minimum threshold of the IC neurons, followed by random sweep sequences including 561 pure tones (50 ms duration) at an interval of 250 ms between each tone pip; in this set-up, the frequency ranged from 1 kHz to 60 kHz with 5.9 octaves, and sound intensities were randomly ranged from 10 to 60 dB SPL with 11 linear steps on the intensity curves. 2) Repetitive stimulation sequences were used for determination of neuronal discharge patterns at CF 40 dB above threshold; this was repeated 100 times with a stimulation interval of 600 ms and duration of 200 ms.

### Single neuron recording

Extracellular electrophysiological recordings were performed in an anechoic chamber where the anesthetized guinea pigs were fixed in place with both ears in a natural unblocked state, which allowed all data obtained in the IC to be binaural. The speakers were placed next to each ear 20 cm from lambda. Single tungsten electrodes (Model 575300, A-M Systems, Sequim, WA, USA), with tip diameters of 50 microns, were controlled by micro-thrusters. Each electrode track through the IC was continued until a reduction in field potential amplitude was observed. The electrode was then immediately withdrawn and repositioned and a new track was started. The raw signal was sampled at 25 kHz, amplified (X 5000; A-M systems, Sequim, WA, USA), and filtered (0.3 ~ 3 kHz; Tucker-Davis Technologies, TDT Co., Alachua, FL, USA). The FRA, CF and threshold at CF of a single IC neuron were determined audio-visually after a sweep sequence was used. Spontaneous activities of the IC neuron were identified during this recording process, following the criteria: firing rate ≥ 5 spikes/s, duration ≥ 10 s. Data were analyzed with OpenSorter software 2.5.0 (Tucker-Davis Technologies, TDT Co., Alachua, FL, USA). The typical IC neurons with spontaneous activities were detected according to the FRAs and the post stimulus time histogram (PSTH) as described previously [10,19].

### Nuclear DNA staining of cochlear hair cell

DNA fluorescent dye Hoechst 33342 (Sigma, St. Louis, USA) was used for the nuclear staining of the Organ of Corti [26]. Hoechst was prepared as 0.04% (wt/vol) stock solution using distilled water and stored in the dark at 4°C. For staining, the stock solution was diluted to 1:200 by phosphate buffered saline (PBS, Beijing Puboxin BioTech Ltd., Beijing, China). After signal recording in IC cells, the animal was sacrificed. The temporal bone was removed along with the opening of the oval window and round window of the ear. A solution of 4% paraformaldehyde was repeatedly infused into the cochlea through both windows. Then the cochlea was quickly dissected from the surrounding bones, and the basilar membrane was isolated, being placed in Hoechst staining solution for 10 minutes in the dark at room temperature. The specimen was washed in the PBS and mounted using 50% glycerol (Beijing Puboxin BioTech Ltd., Beijing, China) on a slide. The fluorescence excitation wavelength for Hoechst 33342 was 337 nm, and the corresponding emission wavelength was 430 nm. Imaging was performed using an Olympus confocal microscope (FluoView FV1000, Olympus China Ltd., Beijing, China), which can be used to count the number of hair cells in the randomly selected areas.

The total number of nuclei in either inner or outer hair cells was counted from three 1-mm sections in length located within the cochlea duct. For the control group, we used 12 cochleae from six guinea pigs. Similarly, for the long recovery group, 12 cochleae from six animals were also examined. The three sites were chosen at 14.5–15.5, 10.5–11.5, and 4.0–5.0 mm from the basal end of the basilar membrane, respectively. The nucleus condition was determined through visual inspection under microscopy.

### Statistical analysis

All results were presented as mean ± standard error (mean ± SE). Comparisons of constituent proportions among the three groups (FRA) were made using PASW Statistics 18.0 software and SPSS 13.0 software (SPSS Inc. IBM Co. Armonk, New York, USA). Comparisons between groups were performed through Student's t-Test, and *P* < 0.05 was considered significant.

## Results

### ABR thresholds in noise exposure group

**Figure 1:**
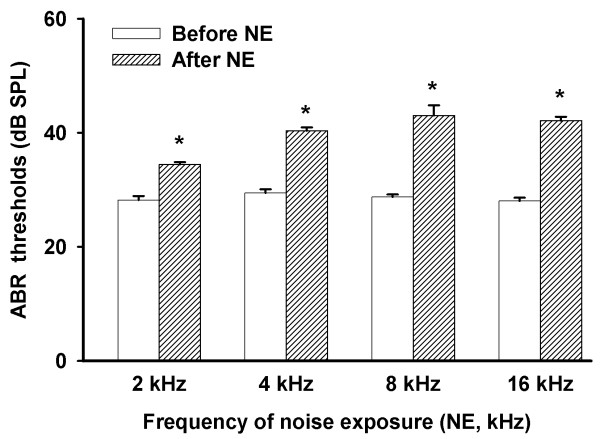
**ABR Thresholds of auditory brainstem response before and after noise exposure (NE).** Statistical data representative of ABR (auditory brainstem response) thresholds before and one day after noise exposure (*n* = 13 from 13 animals) in guinea pigs. Four frequencies were tested: 2 kHz, 4 kHz, 8 kHz, and 16 kHz. *Significant difference from pre-NE group at the same frequency (*P* < 0.01).

Prior to noise exposure, the average auditory brainstem response (ABR) threshold was screened using 2, 4, 8, and 16 kHz. One day after noise overexposure, the ABR thresholds were raised at all the above frequencies compared to pre-exposure levels (*P* < 0.01 from 13 animals). The specific data are listed in Figure 1.

### Nuclear changes of inner and outer hair cells of the cochlea following noise exposure

**Figure 2:**
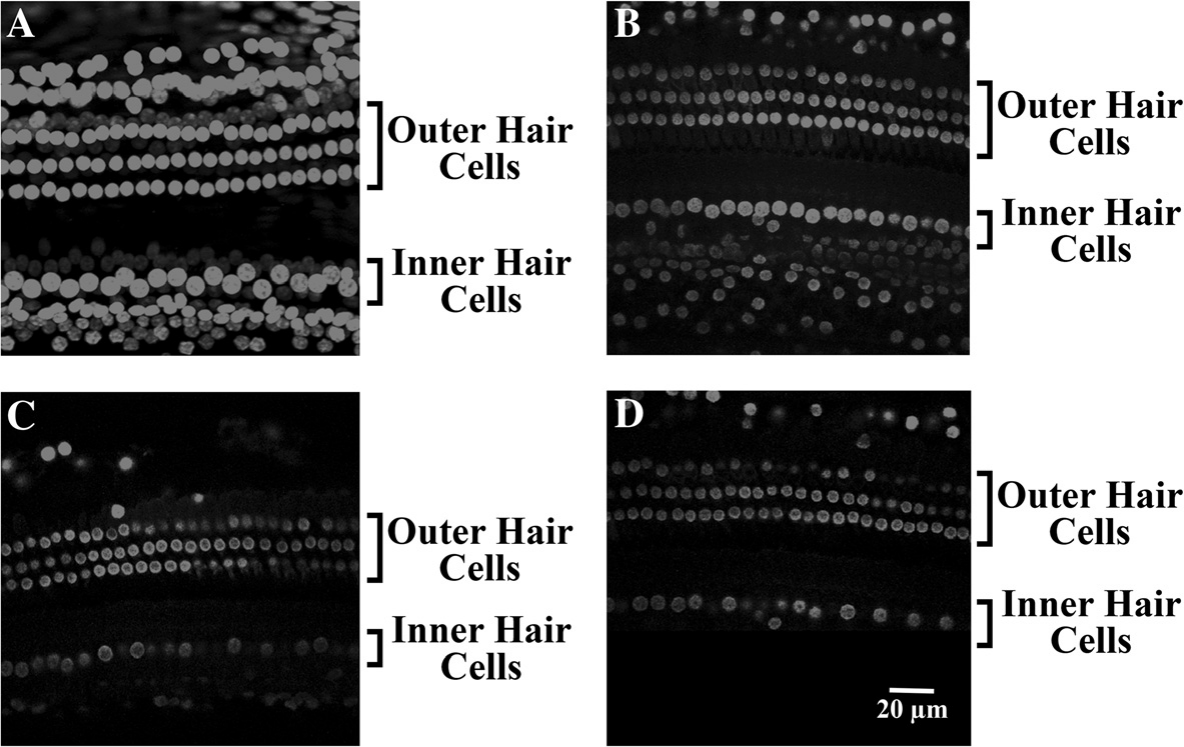
**Representative confocal microscopy (x 40) of fluorescence stained nuclei within inner and outer hair cells in the cochlea of guinea pigs, 11 days after noise exposure (120 dB, 4 hours). A**: Normal levels are observed in the nuclei of inner and outer hair cells of the control subject. The nuclei were arranged normally and no disruption or fragmentations were found. **B**: The image location is in the third turn of the cochlea after noise exposure, where signs of fragmentation equally occurred throughout both inner and outer hair cells. **C**: In the second turn of the cochlea, after noise exposure, more disruption and fragmentation appears to occur in the inner and outer hair cell nuclei compared to the third turn of the cochlea. **D**: The first turn in the cochlea, after noise exposure, exhibited severe disruption and fragmentation compared to the second and third turns in the inner and outer hair cell nuclei.

During the long recovery after noise overexposure, we found significant portions of fragmentation or disappearance of the nuclei within both outer and inner hair cells of the cochlea, as compared to control; for inner hair cells, irregular shape and disorganized alignment of the nuclei were observed (Figure 2A-D). Furthermore, it appears that there are more considerable nuclear disruptions in the 1st and 2nd turn of the cochlea compared to the 3rd turn, suggesting more high frequency damages (in 1st and 2nd turns) than low frequency damage (3rd turn). It has been noted that Figure 2A is a typical picture represented for 1st, 2nd, or 3rd turn in control cochlea (no NE), because in our confocal settings, there was no marked difference among the images taken from these cochlear sections. Thus, Figure 2A was used as a general image taken from the 1st to 3rd turns.

**Figure 3:**
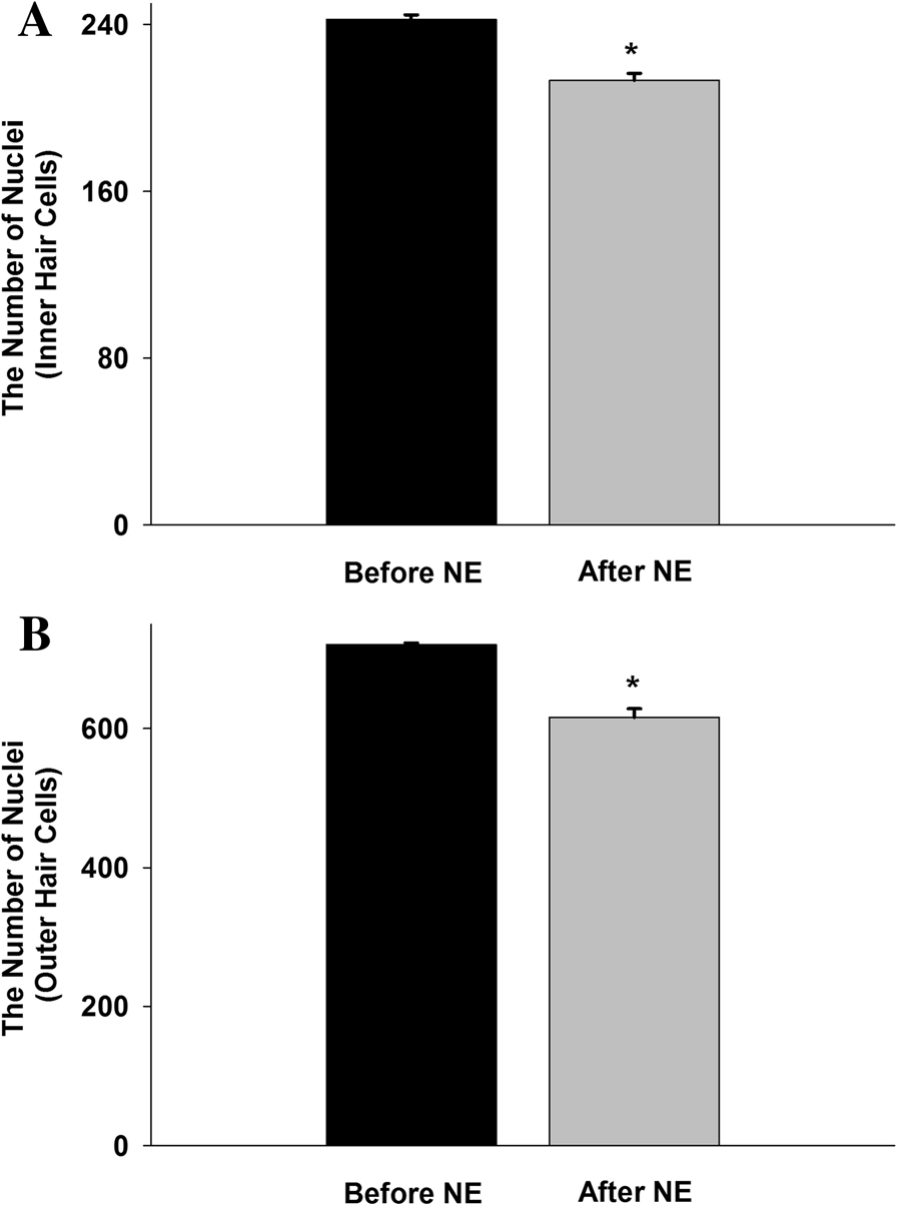
**The number of nuclei in the cochlea in inner (A) and outer (B) hair cells.** Before noise exposure (NE) *vs.* after NE (*n* = 12 from 6 animals; * *P* < 0.05).

As shown in Figure 3, the number of cochlea nuclei in the after-noise exposure (NE) group was significantly reduced compared to pre-NE group (213.1 ± 3.5 *vs.* 242.5 ± 2.1 for inner hair cells; 615.9 ± 12.5 *vs.* 720.9 ± 12.5 for outer hair cells; *n* = 12, *P* < 0.05).

### Composition change of FRA

**Figure 4:**
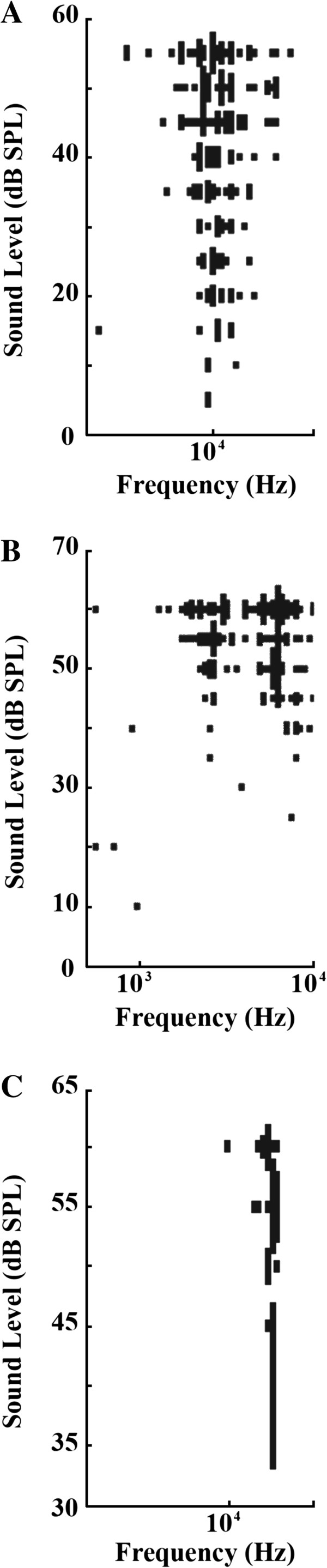
**Representative graphs of frequency response patterns that are the basis of FRA types. (A)** V-type, **(B)** M-type, **(C)** and N-type FRAs created by testing neuronal responses through electrode measurement. Variables included sound frequency and intensity.

**Figure 5:**
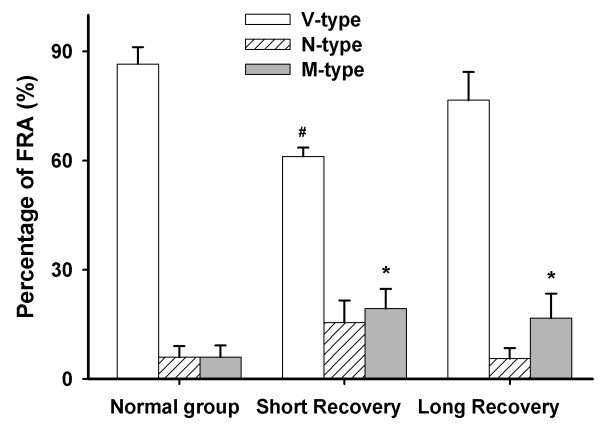
**The percentage of FRA types including V-, N- and M-types in normal, short recovery, and long recovery groups.** # *P* < 0.05 compared to normal groups in V-type (*n* = 6 from 6 animals for normal and *n* = 7 from 7 animals for short recovery). * *P* < 0.05 compared to normal groups in M-type (*n* = 6 from 6 animals for normal, *n* = 7 from 7 animals for short recovery, and *n* = 6 from 6 animals for long recovery).

The description of the FRA follows the methods of Hernandez et al. [19]. The FRA types of recorded neurons from all guinea pigs we used were similar to those previously reported [19]. The majority of FRAs have a single peak, the V-type, as defined by Hernandez et al. [19]. In order to compare the V-type to other types, the double peak or multi-peak type was defined as M-type, and the elongated narrow rectangular band was defined as N-type (Figure 4). As shown in Figure 5, during a short recovery period after noise overexposure, the percentage of V-type was significantly lower than the normal group (61.1 ± 2.4% *vs.* 86.6 ± 4.7%, *n* = 6–7, *P* < 0.01). During the long recovery period, the percentage of V-type shifted back towards normal (76.6 ± 7.8% *vs.* 86.6 ± 4.7%, *n* = 6, n.s.). The percentage of M-type is significantly increased during both short (19.3 ± 5.4%, *n* = 7, *P* < 0.05) and long recovery groups (16.7 ± 6.7%, *n* = 6, *P* < 0.05) compared to the normal group (6.0 ± 3.1%, *n* = 6). However, the percentage of N-type remained constant in both short and long recovery periods (Figure 5).

### Changes of characteristic frequency (CF) over depth

**Figure 6:**
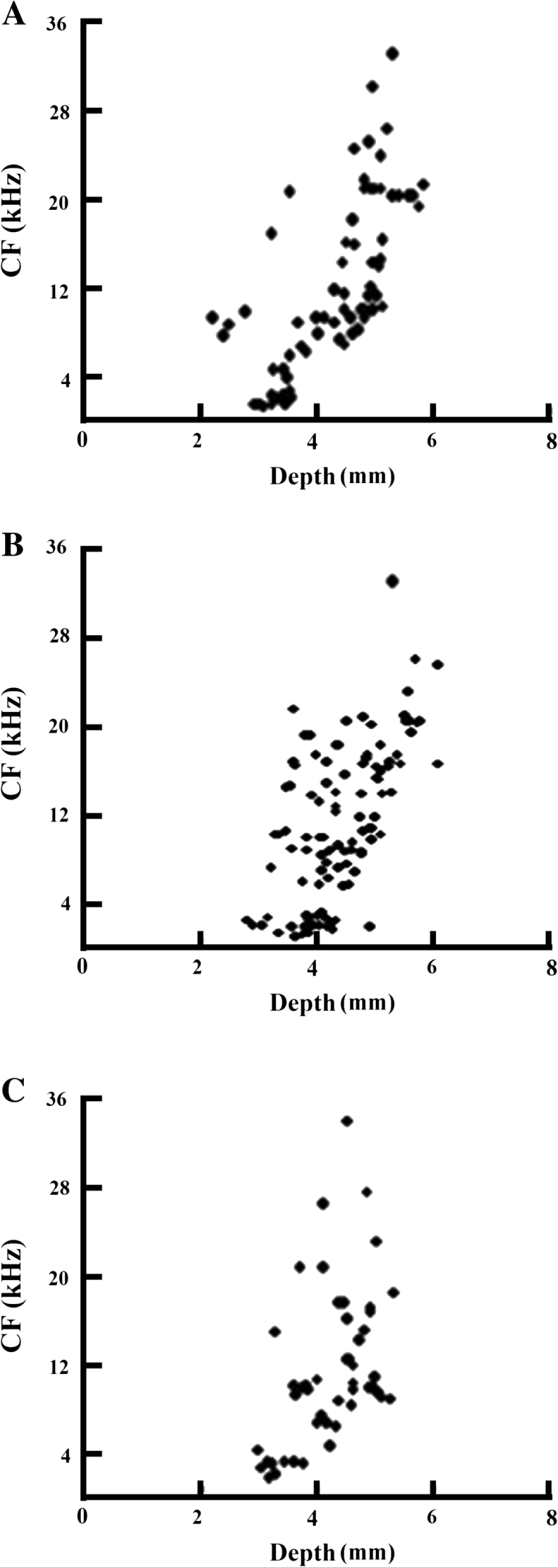
**Function curves providing a correlation between CF (characteristic frequency) and protrusion depth within the IC. A**: A normal distribution can be observed within the control CF map. CF and depth have a positive trend, resulting in higher frequencies the deeper the protrusions within the IC. **B**: Short recovery group shows more of a randomized pattern with dispersed responses above and below 4 kHz creating a gap specifically at that frequency. **C**: Long recovery group exhibited the same type of gap at 4 kHz, but at a reduced level showing signs of recovery.

As shown in Figure 6A, the neuronal CF increased with the increasing depth of the tungsten electrode insertion along the dorsoventral axis of the IC in normal groups (114 neurons from 6 animals). Interestingly, we have observed that for the short recovery group, there is a noticeable gap at 4 kHz (161 neurons from 7 animals, Figure 6B, *P* < 0.01, neurons with 4 kHz gap in short recovery group *vs.* neurons with 4 kHz gap in normal group, post hoc contrast). However, this gap was partially reduced in the long recovery group (74 neurons from 6 animals, *P* < 0.01, neurons with 4 kHz gap in long recovery group *vs.* neurons with 4 kHz gap in normal group; *P* < 0.05 neurons with 4 kHz gap in long recovery group *vs.* in neurons with 4KHz gap in short recovery group, post hoc contrast) (Figure 6C).

### Changes of IC neurons with spontaneous firing

**Figure 7:**
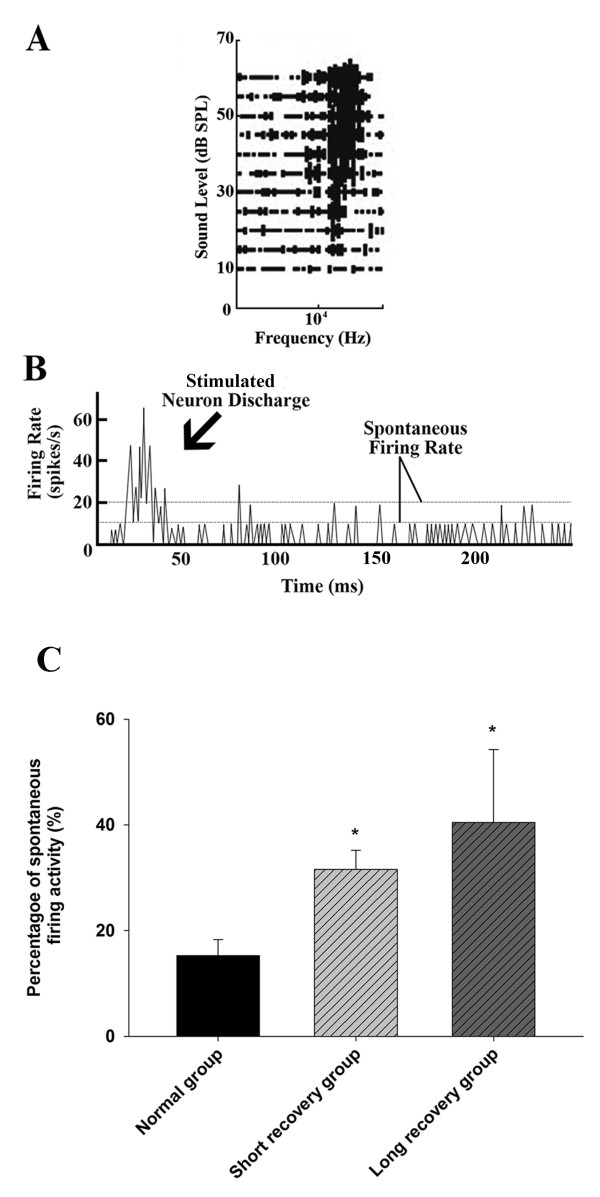
**Spontaneous firing activities in IC neurons. A**: Representative FRA showing typical spontaneous firing. **B**: A typical spontaneous firing curve over time in an IC neuron. **C**: The percentage of neurons with spontaneous firing activity in normal (114 neurons from 6 animals), short recovery (161 neurons from 7 animals) and long recovery groups (74 neuron from 6 animals). *Significant difference from normal group (*P* < 0.05).

Representative FRA with typical spontaneous firing activity (A), corresponding spontaneous firing curve (B), and the percentage of neurons with spontaneous firing activity in different groups (C) are illustrated in Figure 7. Spontaneous activities in the neuron were identified following the criteria: firing rate ≥ 5 spikes/s, duration longer than 10 s. Thus, any firing rate lower than 5 spikes/s was regarded as the absence of spontaneous activities. Specifically, in Figure 7C, the percentage of IC neurons with spontaneous firing activity in the short recovery group was significantly increased compared to normal group (31.6 ± 3.6% *vs.* 15.3 ± 3.0%, *n* = 6-7, *P* < 0.05). The percentage of IC neurons showing spontaneous activity in the long recovery group was also higher than normal group (40.5 ± 13.8% *vs.* 15.3 ± 3.0%, *n* = 6, *P* < 0.05). There was no statistical difference between the short and long recovery groups.

## Discussion

In this study, noise exposure caused two pathological consequences: one is FRA constituent proportions which were noticeably changed; the other is the IC cells which showed higher spontaneous firing activities. We also observed that average ABR thresholds at 4 kHz, 8 kHz, and 16 kHz were ~10 dB higher in the acute noise exposure group. In addition, images of nucleus staining in the hair cells clearly showed extensive disruption and fragmentations in both inner and outer hair cells in the 1st and 2nd turns after NE. However, we are not clear whether the cells were undergoing inflammation or apoptosis. Thus, we only speculate that the cells could possibly be in these stages, which requires further studies.

Consistent with previous studies, narrow-band frequency noise, or pure tone could cause a diffused elevation of CAP thresholds in adjacent frequencies which are normally higher than the exposure frequency. This may be due to the basalward shift of damage to inner and outer hair cells located on the basilar membrane [2,27,28]. According to Seki’s study, hearing loss of more than 10 dB can cause changes of auditory central CF maps in cats [12,15,17]. Moreover, in our study, the underlying causes of the histogram changes (Figure 5) may be complex. FRA constituent proportions’ changes may be induced by elevation of ABR thresholds. After noise exposure, the neuronal output of the cochlea decreased proportionally along with the increased numbers of damaged inner hair cells [17]. Mechanistically, inner hair cells can accurately convert nano level ciliary beats, caused by sound waves, to electrical energy [29]. Outer hair cells can process the space-time coding through a conversion from mechanical energy to the electrical energy generated by the inner hair cells [29]. Therefore, damage to outer hair cells caused changes of space-time coding for output sound, resulting in a decreased cochlear signal output. Thus, the inhibition/excitation balance of IC neurons became disrupted and new sound space-time coding was reconstructed.

We have found that FRA constituent proportions noticeably changed after noise exposure, which is consistent with previous studies [12,15,17]. Specifically, the aforementioned change was most notable in the short recovery group regarding V- and M-type. The long recovery following noise exposure allowed for the continuous restructuring of FRA types back to normal levels, resulting in reduced differences among proportions of FRA types between normal and long recovery groups. This suggests that IC neurons show signs of self-repair during a prolonged time period. These changes in tuning curves are possibly correlated to the fundamental cause of tinnitus or hyperacusis (oversensitivity) [30], which will be the focus of further research.

In the function curves of CF and depth in our experiments, there is a marked gap at 4 kHz measured in the short recovery group as shown in the CF *vs.* depth map. The reduced gap observed in the long recovery group implied the presence of self-repair processes 11–21 days following noise exposure. Although, when compared to chronic tinnitus, the period of 11–21 day is relatively short, it provides a sufficient timeframe to measure the pathological changes in hearing during this period. Note that the noise overexposure frequency was set at 4 kHz, which correlated with this gap. A former study showed that the maximal inhibitory frequency is normally lower than the major frequency at which tinnitus occurs [31]. Our research suggests that the noise of 4 kHz caused a significant shift at 4, 8 and 16 kHz in ABR thresholds.

Increased spontaneous firing rates and the reorganization of the tonotopic map closely follow sensory deafferentation. This also provides an objective signal for tinnitus when associated with hearing loss [32]. It has been reported that after tone-induced (6 kHz) hearing loss in cats, increased synchrony was largely restricted to regions of the auditory cortex where reorganization of the tonotopic map occurred (6 – 10 kHz) compared to non-reorganized regions [8].

In this study, we observed that the FTM reorganization was more noticeable in the short recovery group than the long recovery group. Both groups also had higher spontaneous firing activities than normal. The reorganization of the FTM is obvious in either recovery period when the increased spontaneous firing activity occurs, suggesting that the firing is a relatively dependent event with FTM reorganization. Interestingly, spontaneous rates are increased in animals with tinnitus [33]. Other research has shown that neural tinnitus can continue for life-long periods after recovery from noise-induced damage, whereas CAP thresholds recover only partially weeks after noise-exposure [3,21,29]. Therefore, we speculate that these enhancements in spontaneous firing within the IC neurons relate to tinnitus models previously described [34]. The mechanism of increased spontaneous firing after noise exposure has not yet been fully understood. However, it is likely related to hyperactivity of the auditory nuclei in the brain stem, or a reduction in the normal suppressive activity of the central auditory cortex on peripheral auditory nerve activity as indicated in previous research [35]. It is possible that the disruption of inhibition/excitation balance in IC may arise from noise induced damage to both inner- and outer-hair cells, resulting in increased spontaneous activities in the IC neurons.

## Conclusion

Our research has determined the response of FRAs following noise overexposure. IC neurons during both short and long recovery periods show increased spontaneous firing activity after noise overexposure, when compared to the normal group. In addition, we found that the spontaneous firing and FTM reorganization are two correlated events. Noise induced damage in both inner- and outer- hair cells should be related to these two phenomena. Our results may be useful to develop potential treatments for related hearing diseases.

## Competing interests

No conflicts of interest, financial or otherwise, are declared by the authors.

## Authors’ contributions

FW, LZ, YS, WG performed the experiments, LZ, FW analyzed experimental data, FW, BH and LL designed the project, LL and DH sponsored and supervised the experiments, LZ, FW and EMR wrote the paper, LZ and EMR made figures, LZ supervised the paper submission, editing, and revision. All authors read and approved the final manuscript.
